# First transcriptomic insight into the working muscles of racing pigeons during a competition flight

**DOI:** 10.1007/s11033-024-09566-7

**Published:** 2024-05-08

**Authors:** Monika Stefaniuk-Szmukier, Tomasz Szmatoła, Agnieszka Pustelnik, Katarzyna Ropka-Molik

**Affiliations:** 1 Department of Animal Molecular Biology, National Research Institute of Animal Production, Krakowska 1, Balice, 32-083 Poland; 2 Department of Animal Reproduction, Anatomy and Genomics, The University of Agriculture in Kraków, Al. Mickiewicza 24/28, Kraków, 30-059 Poland; 3 Center for Experimental and Innovative Medicine, The University of Agriculture in Krakow, Rędzina 1C, Kraków, 30-248 Poland

**Keywords:** Racing pigeons, Sport pigeons, Transcriptome profiling, RNA-seq, Pectoralis muscle

## Abstract

**Background:**

The currently known homing pigeon is a result of a sharp one-sided selection for flight characteristics focused on speed, endurance, and spatial orientation. This has led to extremely well-adapted athletic phenotypes in racing birds.

**Methods:**

Here, we identify genes and pathways contributing to exercise adaptation in sport pigeons by applying next-generation transcriptome sequencing of *m.pectoralis* muscle samples, collected before and after a 300 km competition flight.

**Results:**

The analysis of differentially expressed genes pictured the central role of pathways involved in fuel selection and muscle maintenance during flight, with a set of genes, in which variations may therefore be exploited for genetic improvement of the racing pigeon population towards specific categories of competition flights.

**Conclusions:**

The presented results are a background to understanding the genetic processes in the muscles of birds during flight and also are the starting point of further selection of genetic markers associated with racing performance in carrier pigeons.

**Supplementary Information:**

The online version contains supplementary material available at 10.1007/s11033-024-09566-7.

## Introduction

The currently known homing pigeon is a result of crossing many lines of pigeons and a sharp one-sided selection for flight characteristics focused on speed, endurance, and spatial orientation. The purpose of selection focuses on breeding an extraordinarily motivated to get a home bird, that in the shortest possible time in various weather conditions at an average speed greater than 70 km/h will cover a certain distance to the loft [1].

For over 200 years of sports competition, breeders developed a highly specialised breed of pigeon called Racing Homer, paying great attention to improving their ability to approximate the direction to home from foreign locations defeating hundreds of kilometers, avoiding hazards and coping with unexpected weather conditions [2]. The competition aims to compare bird individuals’ flight performance by the speed of returning to the loft. Contests are held at various distances from, short ranging from 95 km to long exhaustive marathons with more than 700 km to cope [3]. When displaced to an unfamiliar location, homing pigeons apply a spatial navigation system and outstanding physiological adaptations to returning to the loft [4] including cardiorespiratory properties [5], energy expenditure supported by anaerobic and aerobic metabolic pathways with efficient circulatory system and oxygen transport [6, 7].

Avian flight is powered primarily by large pectoralis muscles (*m. pectoralis pars thoracicus* which accounts for up to 11% of total body mass and generates up to 95% of the power used for flight. The large m.pectoralis extends from the sternum, clavicle, and ribs to the humerus, and consists of two anatomical parts, the sternobrachialis, and the thoracobrachialis, separated by an aponeurotic central tendon [8]. The fibre type composition contains mainly IIb type, referred to as fast oxidative (~ 85% in pigeons) adapted to anaerobic glycolytic metabolism [11]. Fibers possessed representative sarcomere structures, however, with shorter resting sarcomere lengths compared to mammalians [8].

The avian flying muscles introduce the most energetically expensive muscle work with the highest mass-specific metabolic rates in vertebrates, in comparison to exercising mammals, flapping is energetically more costly than running [6, 9]. To cope with these demands, several mechanisms have been described. Fuel selection of avian muscles during locomotion supports lipid oxidation, with minimum changes in blood glucose concentrations. Pigeons flying at their maximum rate of O_2_ uptake (V̇O_2_,_max_) and a respiratory quotient (RQ) at 0.73 indicating dependence on fat oxidation [10] whilst mammals exercising at similar V̇O_2_,_max_ uptake reach RQ at 0.9 reflect dependence on carbohydrates and finally induce fatigue, with low muscle glycogen and blood glucose [11]. However, birds maintain a very high plasma glucose concentration (1–2 times) compared to mammals of equal body mass but with no harmful physiological effect. It is believed that endogenous antioxidant mechanisms such as free radical scavenging, DNA protection and uric acid-mediated inhibition of lipid peroxidation help with homeostasis maintenance [12].

The dominant role and large size of the *m.pectoralis*, enable an assessment of adaptation and muscle function tailored to meet the mechanical and energetical requirements of exercising flight, compared to exercising mammals of different body masses and aerobic capabilities [13].

Several studies have been undertaken to find molecular pathways modified in skeletal muscles during exercise for example in humans and horses. It has been established that repeated sets of exercises lead to new basal levels of gene expression [14–16]. The molecular mechanism underlying the genetic adaptation during pigeon flight and training remains poorly understood. Since the sport and breeding of racing pigeons is a profitable business covering areas such as nutrition, supplementation, and genetic markers, the aim of the present study was transcriptome profiling of pigeons *m.pectoralis* muscle, collected from untrained birds and trained birds after competing 300 km competition flight with the use of high throughput RNA-sequencing.

## Material and methods

### Animals and study design

The present study was performed on 13 muscle samples of *m. pectoralis* collected from 13 racing pigeons (*Columba livia*). Samples were collected from adult birds never trained for racing (k; n = 5) and birds after competing in a 300 km race (f; n = 8), who had undergone earlier flight training. All birds were bred, and raised on a private loft owner, at the same location, with the same environmental and feeding conditions. The E group was basketed the day before and transported to the place of release (300 km away from the loft).

The racing group was released at 6 a.m. Upon their return, both groups were sedated and euthanized using the cervical dislocation method. The tissues were secured in liquid nitrogen and stored at −80 °C for further analysis.

The Animal Care and Use Committee of the Institute of Pharmacology, Polish Academy of Sciences in Cracow reviewed the experiment protocol. All procedures were conducted following the guidelines for animal care and by qualified staff.

### *m.pectoralis* whole transcriptome sequencing

The total RNA was isolated using TriReagent (Ambion, Life Technologies) and according to the method described by Chomczyński [17]. The muscle samples were homogenized with the use of zirconium oxide beads (diameter 0.5 mm) in BulletBlender homogenizer (Next Advance Inc., USA). The concentration and quality of RNA were estimated on TapeStation 2200 using RNA Screen Tape (Agilent Technologies, Warsaw, Poland). The samples with RIN (RNA integrity number) ranging from 7.7 to 10.0, were sent to sequencing by a commercial company (CeGat gGmbH) on NovaSeq 6000 (Ilumina) apparatus, and 100 bp (± 50 bp) paired-end reads were generated.

### Data analysis

Demultiplexing of the sequencing reads was performed with Illumina bcl2fasq (2.20). Adapters were trimmed with Skewer version 0.2.2 [18]. The FastQC software (v0.11.5) was applied to quality control (QC). The raw reads were aligned to the *Columba livia* genome (assembly 2.1_Colliv) as a reference, and the whole procedure was followed by alignment parameters from ENCODE3’s STAR-RSEM pipeline. The quantification of genes and transcripts was achieved using htseq-count software. The quality control metrics across aligned reads per sample were obtained using RNA-SeQC software [19]. To detect the differentially expressed genes (DEGs) and to show contrasts between the considered conditions, Bioconductor/R-project package, DESeq2 has been applied [20]. The differentially expressed genes were those which reached adjusted p-value < 0.05 (with the use Benjamini/Hochberg method) and fold change ≥ 1.2.

Significantly expressed genes were subjected to functional annotation and pathways enrichment analysis using KOBAS server v3 with the corrected p-value < 0.05 with the *C.Livia* reference. The enriched analysis of independent GO aspects (molecular function, biological process, cellular component) was performed using the Gene Ontology enrichment analysis tool with implemented PANTHER databases. The significant GO terms were those which exceed FDR (False Discovery Rates) < 0.05 [21].

### Validation of RNA-seq with qPCR

cDNA was synthesized from 0.5 μg of the same total RNA used in RNA sequencing using the High-Capacity RNA-to-cDNA™ Kit (Applied Biosystems) as per the manufacturer’s protocol. qPCR reactions were run on a QuantStudio7 (Applied Biosystems, Life Technologies) in a 10-μl reaction containing 0.5 μl of cDNA template, 5 μl of 2 × SYBR Green Master Mix, 0.3 μl of each primer (10 μmol/μl) and 4.4 μl nuclease-free water. The amplification program consisted of one cycle at 95 °C for 10 s, followed by 40 cycles at 95 °C for 15 s and 55 °C for 34 s. The qPCR reactions for *HSF2BP, ADIPOQ* and *PPARD* gene were performed with three biological replicates. Relative gene expression was normalized to the expression of pigeon *ACTB* and calculated with the 2 − ΔΔCT method [22]. Primer pairs for these genes were designed using Primer3 version 4.0.0 and are listed in Supplementary Table S1. The expression levels of the genes obtained in RNA-seq and qPCR were compared with the Pearson correlation coefficients.

## Results

### Sequencing data

The detailed next-generation sequencing statistics for each library are shown in Supplementary Table S2. On average, 14,564,488 mapped read pairs were identified in the pectoralis muscle of racing pigeons and an average of 689,230 were paired with multiple alignments. An average of 10,954,222 read pairs were annotated to the genome (2.1_Colliv). The presented study was submitted to the international functional genomics database—GEO database (Gene Expression Omnibus; NCBI) and assigned GEO accession number—GSE222537.

### Identification of differentially expressed genes, functional annotation, and pathway analysis

**Fig. 1:**
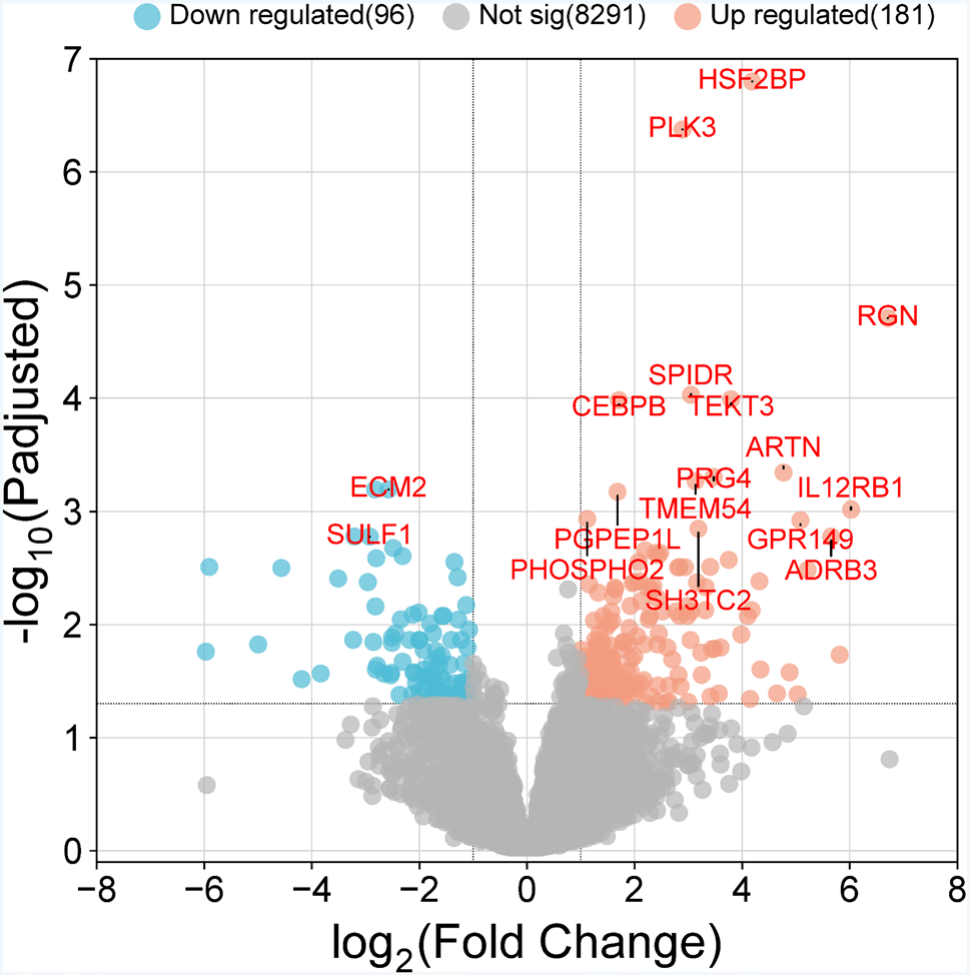
The results of differential gene expression analysis of DEGs. Volcano plots showing the fold change of transcripts between samples and their corrected p values (vertical axis). Dashed grey lines represent the nominal significance threshold (Padj = 0.05)

The muscle transcriptomes of both groups were compared to detect differentially expressed genes under a 300 km competition race condition. The differentially expressed genes (DEGs) selected for further analysis were those which reached adjusted p-value < 0.05 (with the use of the Benjamini/Hochberg method) and fold change ≥ 1.2. With the threshold of padj < 0.05, we identified a total of 502 differentially expressed protein-coding transcripts, of which 103 were identified as novel genes. 330 were up-regulated and 171 were down-regulated (Fig. 1**;** Table 1).

**Table 1:** Top significantly upregulated and downregulated DEG’s before vs 300 km flight

	Gene ID/name	FC	Corrected P-value	Encoded protein function
up	A306_00003776 *RGN*	104.46	1.96E-05	Gluconolactonase, Modulates Ca(2 +) signaling, and Ca(2 +)-dependent cellular processes and enzyme activities
	A306_00011890 *IL12RB1*	65.06	9.61E-04	Interleukin receptor which binds interleukin-12 with low affinity and is involved in IL12 transduction
	A306_00013790 *ADRB3*	50.38	1.66E-03	Beta-adrenergic receptors mediate the catecholamine-induced activation of adenylate cyclase through the action of G proteins. Involved in the regulation of lipolysis and thermogenesis
	A306_00014188 *UBXN11*	37.19	3.29E-03	Involved in the reorganization of actin cytoskeleton mediated and promotes RHOA activation
	A306_00014766 *IQCD*	35.4	5.27E-02	Component of the nexin-dynein regulatory complex (N-DRC), a key regulator of ciliary/flagellar motility which maintains the alignment and integrity of the distal axoneme and regulates microtubule sliding in motile axonemes
	A306_00009404 *GPR149*	33.87	1.19E-03	Transmembrane G protein-coupled receptor
	A306_00003944 *DLX*	32.69	4.13E-02	A transcriptional activator. Plays a role in the terminal differentiation of interneurons
	A306_00004992 *WIF1*	29.48	2.64E-02	WNT Inhibitory Factor 1, binds to WNT proteins and inhibits their activities
	A306_00004091 *ARTN*	27.25	4.56E-04	Ligand for the GFR-alpha-3-RET receptor complex but can also activate the GFR-alpha-1-RET receptor complex
	A306_00014485 *TMEM52B*	25.06	4.06E-02	Transmembrane Protein, located in extracellular exosome
	A306_00011789 *GDF15*	20.24	2.49E-02	Involved in the stress response program of cells after cellular injury. Increased protein levels are associated with disease states such as tissue hypoxia, inflammation, acute injury and oxidative stress
	A306_00007125 *CDHR3*	19.98	4.13E-03	Involved in calcium-dependent cell–cell adhesion via plasma membrane cell adhesion molecules; cell morphogenesis; and cell–cell junction organization
	A306_00009708 *HSF2B*	18.2	1.59E-07	Meiotic recombination factor component of recombination bridges involved in meiotic double-strand break repair
	A306_00004664 *CALCB*	18.08	7.45E-03	Involved in adenylate cyclase-activating G protein-coupled receptor signalling pathway and regulation of cytosolic calcium ion concentration
	A306_00004059 *RAB17*	17.72	4.54E-02	Plays an important role in the regulation of membrane trafficking
	A306_00012620 *HPCA*	17.19	8.37E-03	Calcium-binding proteins that play a role in the regulation of voltage-dependent calcium channels
	A306_00004614 *IFITM10*	15.81	1.22E-02	An integral component of the membrane
	A306_00006451 *TEKT3*	13.86	7.70E-04	Belongs to the tektin family of filament-forming proteins that are coassembled with tubulins to form ciliary and flagellar microtubules
down	A306_00003205 *SLC25A30*	19.8	4.56E-11	Antiporter that transports inorganic anions, sulfite and thiosulfate from the mitochondria, modulating the level of the hydrogen sulfide
	A306_00002582 *COL1A2*	7.08	6.43E-04	The pro-alpha2 chain of type I collagen whose triple helix comprises two alpha1 chains and one alpha2 chain. Type I is a fibril-forming collagen found in most connective tissues
	A306_00010815 *ECM2*	5.95	6.43E-04	Extracellular Matrix Protein 2
	A306_00013554 *LZTS1*	9.25	1.66E-03	Cell-cycle control by interacting with the Cdk1/cyclinB1 complex
	A306_00001417 *SULF1*	7.58	1.66E-03	Exhibits arylsulfatase activity and highly specific endoglucosamine-6-sulfatase activity. It can remove sulfate from the C-6 position of glucosamine within specific subregions of intact heparin
	A306_00006974 *ZNF827*	5.6	2.10E-03	Part of a ribonucleoprotein complex negatively regulates the transcription of genes involved in neuronal differentiation
	A306_00002086 *NPAS3*	6.97	2.58E-03	Member of the basic helix-loop-helix and PAS domain-containing family of transcription factors
	A306_00011062 *TRIM2*	2.55	2.79E-03	UBE2D1-dependent E3 ubiquitin-protein ligase that mediates the ubiquitination of NEFL and phosphorylated BCL2L11
				Plays a neuroprotective function
	A306_00000780 *TPD52L1*	23.69	3.15E-03	Involved in cell proliferation and calcium signalling. It also interacts with the mitogen-activated protein kinase kinase kinase 5 (MAP3K5/ASK1) and positively regulates MAP3K5-induced apoptosis
	A306_00000530 *MTR*	2.44	3.81E-03	Catalyzes the transfer of a methyl group from methylcob(III)alamin (MeCbl) to homocysteine, yielding enzyme-bound cob(I)alamin and methionine in the cytosol
	A306_00005654 *ITGA11*	11.39	3.91E-03	Receptor for collagen. Involved in attaching muscle tissue to the extracellular matrix
	A306_00007045 *SYNPO2*	7.77	4.20E-03	Actin-binding and actin-bundling activity. Induce the formation of F-actin networks in an isoform-specific manner
	A306_00012512 *PLAAT1*	2.19	6.71E-03	Phospholipase A1 (PLA1) and A2 (PLA2) activity, catalyzing the calcium-independent release of fatty acids from the sn-1 or sn-2 position of glycerophospholipids
	A306_00009398 *AADAC*	50.38	7.82E-03	Triglyceride lipase in the liver increases the levels of intracellular fatty acids derived from the hydrolysis of newly formed triglyceride stores and plays a role in very low-density lipoprotein assembly

Next, the significantly expressed genes were subjected to functional annotation and pathways enrichment analysis using KOBAS server v3 with the corrected p-value < 0.05 [21]. The analysis of all significantly expressed DEGs allowed for the detection of 13 significantly deregulated pathways. Among others, the top five significantly overrepresented pathways were autophagy animal, mTOR signalling pathway, lysosome, metabolic pathways, and necroptosis. For top underrepresented pathways were those involved in focal adhesion, ECM-receptor interaction, and Gap junction, respectively (Table 2).

**Table 2:** Significantly overrepresented pathways included DEGs modified in both groups of pigeons

	#Term	Kegg pathway Id	Input number	Background number	Corrected P-value	Input
down	ECM-receptor interaction	clv04512	5	82	0.00605	*FREM2 LAMB3 ITGA11 COL1A2 COL1A1*
	Focal adhesion	clv04510	6	187	0.01757	*PDGFC TLN2 ITGA11 COL1A2 COL1A1 LAMB3*
	Gap junction	clv04540	4	84	0.02467	*TUBAL3 ADCY1 PDGFC TUBB1*
**up**	Phagosome	clv04145	8	119	0.00215	*NCF2 RAB7A CTSL DYNC1I1 ATP6V1H LAMP2 ATP6V1A ATP6V1C1*
	Metabolic pathways	clv01100	27	1303	0.00505	*DOLK PTGS2 ATP6V1A ALG2 ATP6V1H AACS ALG12 UGP2 SMOX BTD DHRS3 UGDH TKT PHOSPHO2 HSD11B2 DPM2 CRYL1 GUK1 ALDOC ALDOB PLA2G4F HYAL2 AKR1D1 GDA B4GALT5 ATP6V1C1 CHPF*
	Autophagy—animal	clv04140	7	119	0.00505	*RRAGD RAB7A GABARAPL1 VMP1 LAMP2 CTSL BCL2*
	N-Glycan biosynthesis	clv00510	4	48	0.02470	*DOLK ALG2 ALG12 DPM2*
	Pentose and glucuronate interconversions	clv00040	3	22	0.02470	*CRYL1 UGDH UGP2*
	Pentose phosphate pathway	clv00030	3	25	0.02470	*ALDOC ALDOB TKT*
	Mitophagy—animal	clv04137	4	55	0.02470	*ATF4 CALCOCO2 GABARAPL1 RAB7A*
	Cytokine-cytokine receptor interaction	clv04060	7	191	0.02470	*BMP4 IL-8 IL4R IL12RB1 IL21R CSF3R GDF15*
	mTOR signaling pathway	clv04150	6	142	0.02470	*RRAGD RPS6KA2 ATP6V1A FNIP1 ATP6V1H ATP6V1C1*
	Necroptosis	clv04217	5	120	0.05272	*CHMP1B BCL2 PLA2G4F MLKL TICAM1*

After a 300 km flight, the gene ontology (GO) enrichment analysis with the use Gene ontology database powered by PANTHER v.17.0 [23], indicates that binding; molecular function and G protein-coupled receptor activity for GO molecular function. GO cellular component processes analysis shows lewy body core, perichromatin fibrils, vacuolar proton-transporting V-type ATPase, V1 domain; and components of vacuolar membrane. The Go biological results pinpoint terms such as chemical homeostasis, regulation of inflammatory response; negative regulation of pri-miRNA transcription by RNA polymerase II; positive regulation of transport, and negative regulation of programmed cell death.

### Validation of DEGs with qPCR

The differential expression of *HSF2BP*, *ADIPOQ*, *PPARD* and *ACTB* genes was validated by qPCR. Pearson’s correlation coefficient calculated between normalized counts obtained after the RNA-seq method and Relative Quantification range 0.43 to 0.97, thereby validating the RNA-seq results.

## Discussion

A sport involving racing pigeons is a highly profitable industry with services such as breeding, feeding, genetic testing, equipment and the starts of pigeons. Pigeons are the only birds competing in sprint flights and analogically to mammalian athletes trained to achieve sufficient fitness. Several studies approached transcriptomic profiling of skeletal muscles before and after different training schedules such as in horses or dogs [16, 24, 25], but to date, no reports reveal results obtained by the use of high throughput methods in birds, under one-sided selection for sport flight performance. The mammalian skeletal muscle response to exercise stimuli results in adaptation through hypertrophy and increasing metabolic capacity manifested by gene expression changes. In this study, samples of the pectoralis major muscle from both untrained and trained racing pigeons, collected after a 300 km flight, were sequenced using Next Generation methods. This approach aimed to explore differentially expressed genes (DEGs) that may reflect not only the effects of flight but also the effects of training. The functional analyses of gene expression profiles in skeletal muscle have identified suites of genes and deregulated molecular pathways that are enriched for functions in energy metabolism, autophagy, mitophagy, necroptosis, lipid and fatty acid metabolism, the mitochondrion, and a wide range of signalling pathways indicate adaptation to prolonged flight at several levels.

In the present study, we found several upregulated transcripts of neural implications such as *NREP* (neuronal regeneration-related protein), *SH3TC2* (SH3 domain and tetratricopeptide repeats-containing protein 2), *ARTN* (artemin), *PRG4* (proteoglycan 4) illustrating adaptation of the neuromuscular junction to exercise [26, 27]. Recent studies have shown that genes involved in the formation of neuromuscular junctions are under positive selection according to the analysis of quantitative traits related to performance in racing pigeons [1, 28, 29]. *SH3TC2* encodes a protein expressed in Schwann cells which are the major glial cell type in the peripheral nervous system. *SH3TC2* interacts with Rab11 GTPase to regulate the recycling of membranes and receptors to the cell surface [30]. The *NREP* gene plays several roles in endocrine signalling, myogenesis, transformation or morphogenesis of neural, muscle and fibroblast cells [31]. *NREP* gene regulates genes associated with lipid synthesis, significantly increases intracellular cholesterol and triglyceride levels, and increases intracellular lipid droplets, supporting the hypothesis of the metabolic shift toward fatty acid utilization during prolonged flight [32]. *PRG4* gene encodes Proteoglycan 4 a protein present at the articular cartilage surface and can regulate the inflammatory response and pain through toll-like receptors (TLRs) during tissue injury [33]. Flying is one of the most energetically challenged endurance exercise among vertebrates. The energetical cost is compared to running or swimming [6]. The respiratory chain capacity of the pigeons’ pectoralis muscle is adequate for the simultaneous maximal rate of respiration with the main use of fatty acids. To cope with fatty acid utilization as the main substrate of energy the entire mechanism for inventory, transportation, and disposal had to be set up.

Within the obtained results, the several genes and metabolic pathways involved in fuel selection towards fatty acids phenotype were significantly expressed. In the presented report, the most significantly downregulated identified gene is *SLC25A30*, encoding member of mitochondrial carrier protein in humans, involved in a shift from carbohydrate to lipid metabolism, and protection from oxidative damage when mitochondrial metabolism increases [34]. Most recent research indicates that the exact role of *SLC25A30* is to transport H_2_S degradation products from the mitochondria thus modulating levels of hydrogen sulfide. Whereas higher concentrations inhibit the electron transport chain and lower maintain mitochondria’s bioenergetic state [35]. Additionally, we observed the upregulation of *SLC15A4* a proton-coupled amino-acid transporter that mediates the transmembrane transport of L-histidine and di-and tripeptides whose activity is pH—dependent and maximized in the acid environment. This strongly supports the switch to fatty acid oxidation. The acidic environment within muscles during the flight is possibly maintained by a set of upregulated genes, involved in the inhibition of lactate dehydrogenase-A (*LDHA*) activity such as; *FNIP2*; *FNIP1*; *ATP6V1A*; *ATP6V1C1*, which are involved in the regulations of folliculin. In turn, folliculin acts as a critical link between mTORC1 with *PPARGC1A*-driven mitochondrial biogenesis, *FLCN* deficiency increased *PPARGC1A* expression and increased mitochondrial function and oxidative metabolism suggests that *FLCN* may play a role in cellular energy and nutrient sensing through interaction with the AMPK–mTOR pathway [36]. Furthermore, *FLCN* controls the movement of the *LDHA* active-site loop regulating its enzyme activity [37]. In the obtained study, no *LDHA* gene was differentially expressed, which indicates other regulations of anaerobic glycolysis.

One of the most up-regulated DEGs within the set of expressed genes is *RGN* (regucalcin). The *RGN* gene encodes a protein with unique calcium-binding without containing the classic EF-hand motif of the calcium-binding domain. The overexpression of *RGN* enhances glucose utilization and lipid production, which regulates insulin’s effect [38]. Furthermore, has a suppressive impact on protein phosphatase activity and participates in lipid metabolism and insulin resistance, however, in avian skeletal muscle insulin does not affect muscle glucose transport, mainly due to the lack of the *SLC2A4* gene. Obtained results however indicate the highly upregulated glucose transporter *SLC2A11* might compensate for the GLUT4 loss. These findings, however, need to be addressed further.

The skeletal muscle tissue is characterized by effective adaptation to changes in metabolic demand. Our results indicate the autophagy pathway as one of the most significant upregulated during intensive flight, which is crucial for cell homeostasis, maintained by the balance of protein synthesis and degradation. This evolutionarily conserved process of controlled degradation of cellular components, in muscles, is regulated by nutrient availability and energy demand. In turn, another significantly upregulated pathway, the mTOR signalling is a key regulator in the anabolic and catabolic signalling of skeletal muscle mass, resulting in the modulation of muscle hypertrophy and muscle wastage [39]. Within our results, both pathways are merged via, the upregulated *RRAGD* (Ras Related GTP Binding D) gene. *RRAGD* plays, through recruitment of the mTORC1, a crucial role in the cellular response to amino acid availability, regulating anabolic pathways in response to nutrients and physical activity [35].

## Conclusions

The next generation of high-throughput sequencing techniques made it possible to undertake research aimed at understanding the genetic basis of pigeon *m.* *pectoralis* performance during flight. In this study, for the first time, samples of *m.pectorallis* from untrained and trained, after 300 km flight, racing pigeons were collected and sequenced with the use of the Next Generation method. The presented results are the skeleton for the next stages of research to understand the processes in the muscles of both trained and untrained birds and also are the background for a further selection of genetic markers associated with certain traits.

## Supplementary Information

Below is the link to the electronic supplementary material.

Supplementary file1 (DOCX 14 KB)

Supplementary file2 (DOCX 16 KB)

## Data Availability

The presented study was submitted to the international functional genomics database—GEO database (Gene Expression Omnibus; NCBI) and assigned GEO accession number—GSE222537.

## References

[1] Caspermeyer J (2018) Using whole-genome analysis to home in on racing pigeon performance. Mol Biol Evol 35:1296–1296. 10.1093/MOLBEV/MSY064 29688545 10.1093/molbev/msy064

[2] Shapiro MD, Domyan ET (2013) Domestic pigeons. Curr Biol. 10.1016/J.CUB.2013.01.063 23618660 10.1016/j.cub.2013.01.063 PMC4854524

[3] pigeonsfci | Federation Colombophile Internationale. https://www.pigeonsfci.net/. Accessed 6 Mar 2024

[4] Hough GE (2022) Neural substrates of homing pigeon spatial navigation: results from electrophysiology studies. Front Psychol. 10.3389/FPSYG.2022.867939 35465504 10.3389/fpsyg.2022.867939 PMC9020565

[5] Peters GW, Steiner DA, Rigoni JA et al (2005) Cardiorespiratory adjustments of homing pigeons to steady wind tunnel flight. J Exp Biol 208:3109–3120. 10.1242/JEB.01751 16081609 10.1242/jeb.01751

[6] Butler PJ (2016) The physiological basis of bird flight. Philos Trans R Soc B Biol Sci. 371(1704):20150384 10.1098/rstb.2015.0384 PMC4992708 27528774

[7] Scott GR, Milsom WK (2006) Flying high: a theoretical analysis of the factors limiting exercise performance in birds at altitude. Respir Physiol Neurobiol 154:284–301. 10.1016/j.resp.2006.02.012 16563881 10.1016/j.resp.2006.02.012

[8] Renato Pinto J, Mamidi R, Joan Grove T et al (2020) Evolution of flight muscle contractility and energetic efficiency. Front Physiol 11:1038. 10.3389/fphys.2020.01038 33162892 10.3389/fphys.2020.01038 PMC7581897

[9] Evangelista D, Fernández MJ, Berns MS et al (2010) Hovering energetics and thermal balance in anna’s hummingbirds (*Calypte anna*). Physiol Biochem Zool 83:406–413. 10.1086/651460 20350142 10.1086/651460

[10] Rothe HJ, Biesel W, Nachtigall W (1987) Pigeon flight in a wind tunnel: II. Gas exchange and power requirements. J Comp Physiol B Biochem Syst Environ Physiol 157:99–109. 10.1007/BF00702734

[11] Coyle EF (1995) Substrate utilization during exercise in active people. Am J Clin Nutr 61:968S-979S. 10.1093/AJCN/61.4.968S 7900696 10.1093/ajcn/61.4.968S

[12] Braun EJ, Sweazea KL (2008) Glucose regulation in birds. Comp Biochem Physiol—B Biochem Mol Biol 151:1–9. 10.1016/J.CBPB.2008.05.007 18571448 10.1016/j.cbpb.2008.05.007

[13] Biewener AA (2011) Muscle function in avian flight: achieving power and control. Philos Trans R Soc B Biol Sci 366:1496–1506. 10.1098/rstb.2010.0353 10.1098/rstb.2010.0353 PMC3130450 21502121

[14] Lindholm ME, Giacomello S, Werne Solnestam B et al (2016) The impact of endurance training on human skeletal muscle memory, global isoform expression and novel transcripts. PLoS Genet 12(9):e1006294 27657503 10.1371/journal.pgen.1006294 PMC5033478

[15] McGivney BA, McGettigan PA, Browne JA et al (2010) Characterization of the equine skeletal muscle transcriptome identifies novel functional responses to exercise training. BMC Genom. 10.1186/1471-2164-11-398 10.1186/1471-2164-11-398 PMC2900271 20573200

[16] Ropka-Molik K, Stefaniuk-Szmukier M, Żukowski K et al (2017) Exercise-induced modification of the skeletal muscle transcriptome in Arabian horses. Physiol Genom. 10.1152/physiolgenomics.00130.2016 10.1152/physiolgenomics.00130.2016 28455310

[17] Chomczynski P (1993) A reagent for the single-step simultaneous isolation of RNA, DNA and proteins from cell and tissue samples. Biotechniques 15:532–537 7692896

[18] Jiang H, Lei R, Ding SW, Zhu S (2014) Skewer: a fast and accurate adapter trimmer for next-generation sequencing paired-end reads. BMC Bioinform 15:1–12. 10.1186/1471-2105-15-182/FIGURES/5 10.1186/1471-2105-15-182 PMC4074385 24925680

[19] Deluca DS, Levin JZ, Sivachenko A et al (2012) RNA-SeQC: RNA-seq metrics for quality control and process optimization. Bioinformatics 28:1530. 10.1093/BIOINFORMATICS/BTS196 22539670 10.1093/bioinformatics/bts196 PMC3356847

[20] Love MI, Huber W, Anders S (2014) Moderated estimation of fold change and dispersion for RNA-seq data with DESeq2. Genome Biol. 10.1186/S13059-014-0550-8 25516281 10.1186/s13059-014-0550-8 PMC4302049

[21] Bu D, Luo H, Huo P et al (2021) KOBAS-i: intelligent prioritization and exploratory visualization of biological functions for gene enrichment analysis. Nucleic Acids Res 49:W317–W325. 10.1093/NAR/GKAB447 34086934 10.1093/nar/gkab447 PMC8265193

[22] Van Borm S, Steensels M, Ferreira HL et al (2007) A universal avian endogenous real-time reverse transcriptase-polymerase chain reaction control and its application to avian influenza diagnosis and quantification. Avian Dis 51:213–220. 10.1637/7552-033106R.1 17494556 10.1637/7552-033106R.1

[23] Mi H, Muruganujan A, Ebert D et al (2019) PANTHER version 14: more genomes, a new PANTHER GO-slim and improvements in enrichment analysis tools. Nucleic Acids Res 47:D419–D426. 10.1093/NAR/GKY1038 30407594 10.1093/nar/gky1038 PMC6323939

[24] Park K-D, Park J, Ko J et al (2012) Whole transcriptome analyses of six thoroughbred horses before and after exercise using RNA-Seq. BMC Genom. 10.1186/1471-2164-13-473 10.1186/1471-2164-13-473 PMC3472166 22971240

[25] Brass EP, Peters MA, Hinchcliff KW et al (2009) Temporal pattern of skeletal muscle gene expression following endurance exercise in Alaskan sled dogs. J Appl Physiol 107:605–612. 10.1152/JAPPLPHYSIOL.91347.2008 19498091 10.1152/japplphysiol.91347.2008

[26] Gardiner P, Dai Y, Heckman CJ (2006) Effects of exercise training on α-motoneurons. J Appl Physiol 101:1228–1236. 10.1152/JAPPLPHYSIOL.00482.2006 16778002 10.1152/japplphysiol.00482.2006

[27] Deschenes MR (2019) Adaptations of the neuromuscular junction to exercise training. Curr Opin Physiol. 10.1016/j.cophys.2019.02.004

[28] Gazda MA, Andrade P, Afonso S et al (2018) Signatures of selection on standing genetic variation underlie athletic and navigational performance in racing pigeons. Mol Biol Evol. 10.1093/molbev/msy030 29547891 10.1093/molbev/msy030

[29] Shao Y, Tian HY, Zhang JJ et al (2020) Genomic and phenotypic analyses reveal mechanisms underlying homing ability in pigeon. Mol Biol Evol 37:134–148. 10.1093/MOLBEV/MSZ208 31501895 10.1093/molbev/msz208

[30] Stendel C, Roos A, Kleine H et al (2010) SH3TC2, a protein mutant in charcot-marie-tooth neuropathy, links peripheral nerve myelination to endosomal recycling. Brain 133:2462–2474. 10.1093/brain/awq168 20826437 10.1093/brain/awq168

[31] Taylor GA, Hudson E, Resau JH, Vande Woude GF (2000) Regulation of P311 expression by met-hepatocyte growth factor/scatter factor and the ubiquitin/proteasome system. J Biol Chem 275:4215–4219. 10.1074/JBC.275.6.4215 10660586 10.1074/jbc.275.6.4215

[32] Leung JK, Cases S, Vu TH (2008) P311 functions in an alternative pathway of lipid accumulation that is induced by retinoic acid. J Cell Sci 121:2751–2758. 10.1242/JCS.027151 18664493 10.1242/jcs.027151 PMC2975553

[33] Iqbal SM, Leonard C, Regmi SC et al (2016) (2016) Lubricin/proteoglycan 4 binds to and regulates the activity of toll-like receptors in vitro. Sci Reports 61(6):1–12. 10.1038/srep18910 10.1038/srep18910 PMC4707532 26752378

[34] Haguenauer A, Raimbault S, Masscheleyn S et al (2005) A new renal mitochondrial carrier, KMCP1, is up-regulated during tubular cell regeneration and induction of antioxidant enzymes. J Biol Chem 280:22036–22043. 10.1074/JBC.M412136200 15809292 10.1074/jbc.M412136200

[35] Paul BD, Snyder SH, Kashfi K (2021) NC-ND license effects of hydrogen sulfide on mitochondrial function and cellular bioenergetics. Redox Biol. 10.1016/j.redox.2020.101772 33137711 10.1016/j.redox.2020.101772 PMC7606857

[36] Hasumi H, Baba M, Hasumi Y et al (2012) Regulation of mitochondrial oxidative metabolism by tumor suppressor FLCN. J Natl Cancer Inst 104:1750–1764. 10.1093/JNCI/DJS418 23150719 10.1093/jnci/djs418 PMC3502196

[37] Woodford MR, Baker-Williams AJ, Sager RA et al (2021) The tumor suppressor folliculin inhibits lactate dehydrogenase a and regulates the Warburg effect. Nat Struct Mol Biol 28:662–670. 10.1038/s41594-021-00633-2 34381247 10.1038/s41594-021-00633-2 PMC9278990

[38] Nakashima C, Yamaguchi M (2006) Overexpression of regucalcin enhances glucose utilization and lipid production in cloned rat hepatoma H4-II-E cells: involvement of insulin resistance. J Cell Biochem 99:1582–1592. 10.1002/JCB.21005 16817230 10.1002/jcb.21005

[39] Yoon MS (2017) mTOR as a key regulator in maintaining skeletal muscle mass. Front Physiol 8:788 29089899 10.3389/fphys.2017.00788 PMC5650960

