# Supplementary material for: First transcriptomic insight into the working muscles of racing pigeons during a competition flight
Source: Mol Biol Rep. 2024 May 8;51(1):625. doi: 10.1007/s11033-024-09566-7 (PMC11078782; doi:10.1007/s11033-024-09566-7)
Supplement: Supplementary file 1 — Supplementary file1 (DOCX 14 KB) [file 11033_2024_9566_MOESM1_ESM.docx]

Table S1. Primers sequences for target and control genes used for RNA-seq validation by qPCR.

| **Gene number** | **Gene name** | **Primers sequence (**5'🡪3'**)** | **Product size (bp)** |
| --- | --- | --- | --- |
| ***A306_00009708*** | *HSF2BP* | F: CGCAATGATACGCTGAAATG | 172 |
|  |  | R: TCCTTTGCTTTGGTGGCTAT |  |
| ***A306_00013054*** | *PPARD* | F: CCTTCAGTGGACCTAAAAGGA | 180 |
|  |  | R: CCGCCACAAGACACAGTAGA |  |
| ***A306_00013861*** | *ADIPOR1* | F: TCCTTTGCTTTGGTGGCTAT | 229 |
|  |  | R: GAGGAAGAGGAGGAGGTGGT |  |
|  |  | R: CAGGTGGGTGTCCCAGTC |  |
| ***A306_00009109*** | *ACTB* | F: GTGGATCAGCAAGGAGT | 174 |
|  |  | R: TCATCAAGGTGTGGGTG |  |
