# Supplementary material for: First transcriptomic insight into the working muscles of racing pigeons during a competition flight
Source: Mol Biol Rep. 2024 May 8;51(1):625. doi: 10.1007/s11033-024-09566-7 (PMC11078782; doi:10.1007/s11033-024-09566-7)
Supplement: Supplementary file 2 — Supplementary file2 (DOCX 16 KB) [file 11033_2024_9566_MOESM2_ESM.docx]

|  | *HSF2BP* | | *ADIPOQ* | | *PPARD* | |
| --- | --- | --- | --- | --- | --- | --- |
| sample | Normalized counts | RQ | Normalized counts | RQ | Normalized counts | RQ |
| f1 | 21,44 | 1,00 | 870,27 | 1,00 | 1235,53 | 1,00 |
| k2 | 2,21 | 1,35 | 740,39 | 1,96 | 1599,02 | 2,17 |
| k3 | 15,12 | 1,77 | 891,86 | 2,55 | 1163,95 | 2,46 |
| k4 | 8,32 | 1,07 | 798,36 | 1,47 | 1459,50 | 2,33 |
| k5 | 26,87 | 2,87 | 846,97 | 2,59 | 2203,59 | 3,19 |
| f6 | 402,34 | 12,18 | 722,23 | 7,19 | 2768,71 | 2,99 |
| f7 | 133,58 | 6,90 | 755,80 | 8,38 | 4824,46 | 5,61 |
| f8 | 155,14 | 6,47 | 718,61 | 2,77 | 4073,36 | 9,00 |
| f9 | 309,84 | 16,33 | 738,44 | 1,29 | 9943,55 | 7,05 |
| f10 | 118,44 | 3,92 | 864,47 | 1,71 | 2370,73 | 3,32 |
| f11 | 223,21 | 9,72 | 689,28 | 6,92 | 9473,95 | 13,69 |
| k12 | 16,07 | 2,43 | 886,16 | 4,07 | 1633,02 | 3,39 |
| f13 | 645,50 | 17,44 | 1215,98 | 1,39 | 2473,42 | 2,07 |
|  |  |  |  |  |  |  |
| r2 |  | 0,97 |  | 0,43 |  | 0,83 |

Table S.2. Results of qPCR validation for RNA-seq experiment.
